# Glucocorticoid-mediated acetylated regulation of glucocorticoid receptor and Histone3/Histone4 influence glucocorticoid heterogeneity in children patients with primary nephrotic syndrome

**DOI:** 10.1186/s13052-025-01914-y

**Published:** 2025-03-07

**Authors:** Yu heng Liang, Can Liang, Jin Cheng, Qianqian Peng, Ping Zeng, Fengjun Guan

**Affiliations:** 1 Department of Pediatrics, Affiliated Hospital of Xuzhou Medical University, Xuzhou, Jiangsu 221002 China; 2 Xuzhou Children’s Hospital, Xuzhou, Jiangsu 221002 China; 3 Department of Biostatistics, School of Public Health, Xuzhou Medical University, Xuzhou, Jiangsu 221004 China; 4 Center for Medical Statistics and Data Analysis, Xuzhou Medical University, Xuzhou, Jiangsu 221004 China

**Keywords:** Primary nephrotic syndrome, Glucocorticoid receptor, Acetylation, Histone3, Histone4, Nuclear factor-κB

## Abstract

**Background:**

Glucocorticoid (GC) response heterogeneity has been recognized as an unfavorable prognostic factor, yet the underlying mechanism remains elusive. In this study, we endeavored to investigate the potential causes from an epigenetic perspective.

**Methods:**

The protein expression levels of the glucocorticoid receptor (GR), acetylated GC receptor (Ac-GR), acetylated histone3 (Ac-H3), histone4 (Ac-H4), and the activity of nuclear factor-κB (NF-κB) were quantified in the peripheral blood lymphocytes of patients exhibiting diverse GC responses.

**Results:**

Before GC treatment, the study included 32 children with steroid-sensitive nephrotic syndrome (SSNS) and 15 children with steroid-resistant nephrotic syndrome (SRNS). The expression levels of Ac-GR, Ac-H3, Ac-H4, and NF-κB activity were significantly different among the control, SSNS, and SRNS groups (*p*-values < 0.05). Specifically, the expressions were relatively low in the control group, moderately high in the SSNS group, and significantly elevated in the SRNS group. After GC treatment, the expressions of Ac-GR, Ac-H3, Ac-H4, and NF-κB activity decreased in the SSNS children (mean = 0.397, SD = 0.049, *p* = 4.42E-11 for NF-κB; mean = 0.429, SD = 0.107, *p* = 8.41E-6 for Ac-GR, mean = 0.652, SD = 0.126, *p* = 5.38E-8 for Ac-H3, and mean = 0.599, SD = 0.098, *p* = 1.24E-7 for Ac-H4), while they increased in the SRNS patients (mean = 0.576, SD = 0.064, *p* = 4.53E-5 for NF-κB, mean = 0.498, SD = 0.113, *p* = 8.81E-3 for Ac-GR). The correlations among these expressions differed between the SSNS and SRNS groups. In the SSNS group, a positive correlation was identified between NF-κB (mean = -0.156, SD = 0.090) activity and Ac-GR (mean = -0.148, SD = 0.157) protein expression after GC treatment (*r* = 0.392, *p* = 0.026), whereas a negative correlation was observed in the SRNS group (mean = 0.195, SD = 0.130 for NF-κB, mean = 0.173, SD = 0.221 for Ac-GR, *r* = -0.367, *p* = 0.178). Additionally, a positive correlation for the difference between Ac-H3 and Ac-H4 expressions was observed in the SSNS group (mean = -0.239, SD = 0.190 for Ac-H3, mean = -0.203, SD = 0.168 for Ac-H4, *r* = 0.394, *p* = 0.026), which was absent in the SRNS group.

**Conclusion:**

The expression levels of Ac-GR, Ac-H3, and Ac-H4 differed significantly among children’s patients with primary nephrotic syndrome (PNS) who manifested distinct GC responses. It is suggested that GC therapy may has a direct impact on the acetylation of GR, H3, and H4.

## Background

Although glucocorticoid (GC) exhibits significant efficacy in children PNS patients for therapeutic purposes, a subset of these individuals who receive GC therapy may presented as GC insensitivity. Approximately 25% of children with PNS and GC resistance can be ascribed to genetic factors [1]. Notably, the majority of children with steroid-resistant nephrotic syndrome (SRNS) become steroid-sensitive after the administration of second-line immunosuppressive drugs, indicating that a significant proportion of GC resistance cannot be accounted for by an abnormal genetic background.

Recent investigations have revealed an escalating incidence of primary GC resistance among PNS patients [2]. The mechanism underlying GC resistance potentially encompasses both genetic factors and environmental influences or medication regimens during the disease progression. GC response alterations during GC treatment pertain to the epigenetic regulatory mechanism, which is likely one of the principal contributors to late-onset GC resistance, also referred to as secondary GC resistance [3]. Genetic pharmacological studies have demonstrated that the variability in individual GC responses to diseases and the development of GC resistance following GC administration are associated with epigenetic factors [4–6]. A plethora of previous studies have illustrated that epigenetic regulation plays a crucial role in drug responses, such as those to GC [7, 8], with histone modification being the predominant modality within this regulatory framework [9]. It has been established that histone acetylation-induced chromosomal structural modifications can precipitate differential drug responses.

GC exerts its function through binding to the glucocorticoid receptor (GR) [10, 11]. GR is capable of deacetylating histones and modulating the transcription of inflammatory genes. Subsequently, by recruiting and activating histone deacetylases (HDACs), it suppresses inflammation. However, in children’s patients with PNS, this process may lead to GC resistance. It has been reported that histone acetylation is also implicated in GC resistance in patients with chronic obstructive pulmonary disease (COPD) and asthma [12, 13], as a result of decreased HDAC activity leading to a reduction in the level of deacetylated histones. Our prior study identified an association between the decreased level of deacetylated histone2 (HDAC2) and GC resistance in PNS patients [14].

Evidence indicates that post-translational modifications of proteins, particularly histone modification, play a crucial role in GC response [15]. GC modifies the chromatin structure by altering the expression of inflammatory genes [16]. Histone acetylation is a dynamic and reversible process regulated bidirectionally by histone acetylation transferase (HAT) and histone deacetylase (HDAC). Since histone acetylation is associated with gene activation, the removal of acetyl groups by HDACs leads to chromatin remodeling and subsequent gene repression or silencing. GC binds to GR in the cytoplasm, translocates to the cell nucleus after acetylation, interacts with NF - κB for the anti - inflammatory process, and GR may have other epigenetic changes affecting its response to GC via ligand binding [17, 18]. Histone deacetylase-2 (HDAC2) is an essential component of the GR-corepressor complex, which mediates the trans-repression of NF-κB transcriptional activity by deacetylating histones in the promoters of pro-inflammatory factor-encoding genes. The deacetylated GR can suppress the activity of NF-κB through protein-protein interactions without altering the DNA structure [19].

Hence, we hypothesize that aberrant histone acetylation may be involved in GC resistance in PNS patients. Prompted by these observations, the primary aim of this study is to elucidate the mechanism by which histone acetylation participates in GC resistance in PNS children.

## Methods

### Subjects

Children’s patients with PNS at the initial onset were recruited. According to the criteria established by the International Study of Kidney Disease in Children (ISKDC) [20], PNS was characterized by nephrotic-range proteinuria (≥ 40 mg/m²/hour or urine protein/creatinine ratio ≥ 200 mg/mL or 3 + protein on urine dipstick), hypoalbuminemia (< 25 g/L), and edema. The following exclusion criteria were applied during patient selection: (i) children under 1 year of age were excluded to rule out congenital and infantile nephrosis; (ii) those who had received neither GC nor immunosuppressant within the three months prior to study enrollment; (iii) individuals with a history of recent infections, trauma, psychological stress, or radiation exposure. Based on these criteria, a total of 47 children with PNS were ultimately incorporated into the study. Subsequently, based on their responses to GC therapy during the six-week follow-up period, they were categorized into two groups: the steroid-sensitive nephrotic syndrome (SSNS) group and the steroid-resistant nephrotic syndrome (SRNS) group. The SSNS patients were defined as those who exhibited negative urine protein within six weeks of treatment and maintained this status throughout the follow-up. The SRNS patients were defined as those with positive urine protein even after six weeks of treatment, with a urinary protein level exceeding 50 mg/kg/d. Additionally, 15 healthy children were recruited as controls, without a history of recent infections, trauma, psychological stress, or radiation exposure.

This research was conducted in the Affiliated Hospital of Xuzhou Medical University from September 2020 to December 2022. The research protocol was reviewed and approved by the institutional review board of the aforementioned hospital prior to the commencement of the study (review number: xyfylw2013036). Written informed consents from the parents of all participants were acquired prior to the initiation of the research.

### Lymphocyte’s separation

For each subject in this study, fasting aseptic venous blood samples were collected at 8 am both prior to and subsequent to a six-week GC treatment. An equal volume of phosphate-buffered saline (PBS) was added to the lymphocyte separation medium (TBD-LTS1077, Tianjin Haoyang Biotechnology Co., China). Subsequently, the samples were centrifuged, and lymphocytes were isolated, washed, and finally collected for total protein and RNA extraction. The collected samples were stored in a -80 °C refrigerator until further analysis.

### Western blotting for GR, Ac-GR, Ac-H3, and Ac-H4 expression

Protein extraction was performed in accordance with the instructions provided in the total protein extraction manual (Total Protein Extraction Kit, Applygen Technologies Inc., Doc Rev 0611, Page 1 of 2). The protein concentration was quantified using the bicinchoninic acid (BCA) method (BD0028; the kit was supplied by Biyuntian Co., China). Subsequently, the total protein was subjected to electrophoresis in a 10% sodium dodecyl sulfate-polyacrylamide gel electrophoresis (SDS-PAGE) system. Rabbit anti-human antibodies against GR, Ac-GR, Ac-H3 (provided by Millipore Company, Anti-Acetyl-Histone H3, 06-599), and Ac-H4 (Anti-Acetyl-Histone H4, 06-866, Millipore Company) were added sequentially. The secondary antibody labeled with goat anti-rabbit alkaline phosphatase (1:1000, Bioengineering Co. LTD, Shanghai, China) was incubated at room temperature for 2 h. The bands were scanned using gel image analysis software, and a semi-quantitative analysis was conducted with β-actin serving as the internal reference.

### ELISA for NF-κB activity measurement in serum

The activity of NF-κB in the serum was determined using the human NF-κB activity assay kit (Rapid bi, USA) (the Histone ELISA kit contains Biotin antibody, Biotin antibody Diluent, Standard, Sample Diluent, etc.) following the manufacturer’s instructions precisely. The NF-κB activity was quantified as the ratio of the absorbance at 450 nm after 15 min of incubation at 37℃.

### Statistical analysis

Protein expression levels were presented as mean ± sd (standard deviation). One-way ANOVA was employed to test the differences in protein expressions. To further investigate the potential associations between histone modification and GC response, a mediation analysis was conducted. In this analysis, the GC response (categorized as SRNS or SSNS) was designated as the exposure variable, NF-κB as the mediator variable, and the expressions of Ac-GR (or Ac-H3 or Ac-H4) as the outcome variables. Briefly, in the mediation analysis framework [21–25], the effect of the GC response on the Ac-GR expression without accounting for the influence of NF-κB is defined as the total effect. Conversely, the direct effect of the GC response on the Ac-GR expression is estimated by adjusting for the mediator’s influence. The mediation effect is computed as the product of the effect of the GC response on the NF-κB expression and the effect of the NF-κB expression on the Ac-GR expression, and can be tested using the Sobel method [26]. Under certain regular assumptions, the effects in the mediation analysis can be causally interpreted [23–25]. Throughout this study, the significance level was set at 0.05. All data analyses were carried out in the R statistical computing environment (version 3.5.1).

## Results

### Basic characteristics

The SSNS group comprised 32 children (18 males and 14 females), with an age range of 2 to 12 years and a median age of 5.6 years. The SRNS group consisted of 15 children (10 males and 5 females), with an age range of 2 to 11 years and a median age of 5.8 years.

### Comparison of Ac-GR, Ac-H3, and Ac-H4 expressions and NF-κB activity prior to GC treatment

**Fig. 1:**
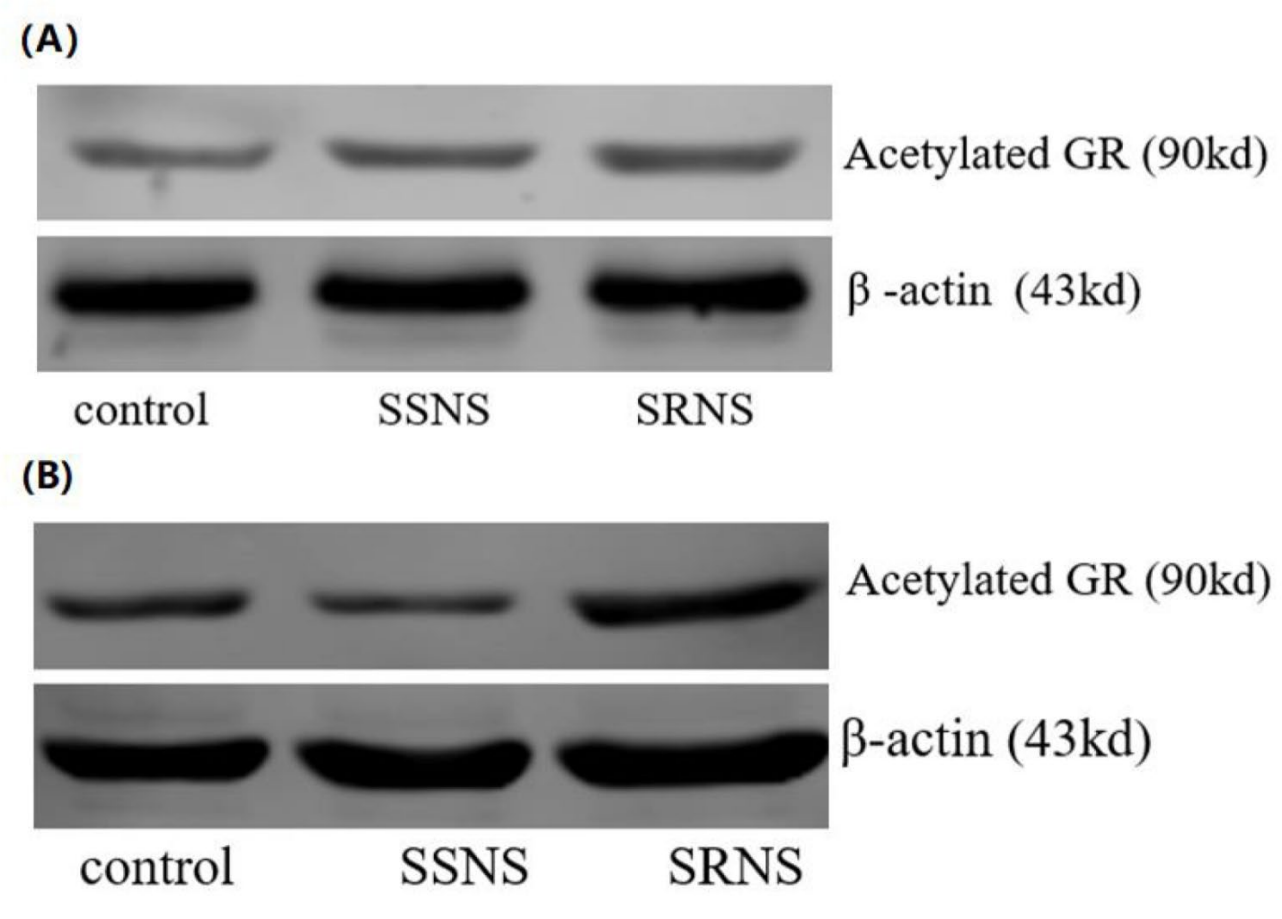
Acetylated GR protein expression before (**A**) and after (**B**) the GC treatment. The acetylated GR protein expression in the SSNS group and the control group is lower than that in the SRNS group before the GC treatment. The acetylated GR protein expression in the SSNS group and the control group is also lower than that in the SRNS group after the GC treatment. GR: glucocorticoid receptor; GC: glucocorticoid; SSNS: steroid sensitive nephrotic syndrome; SRNS: steroid resistant nephrotic syndrome

**Fig. 2:**
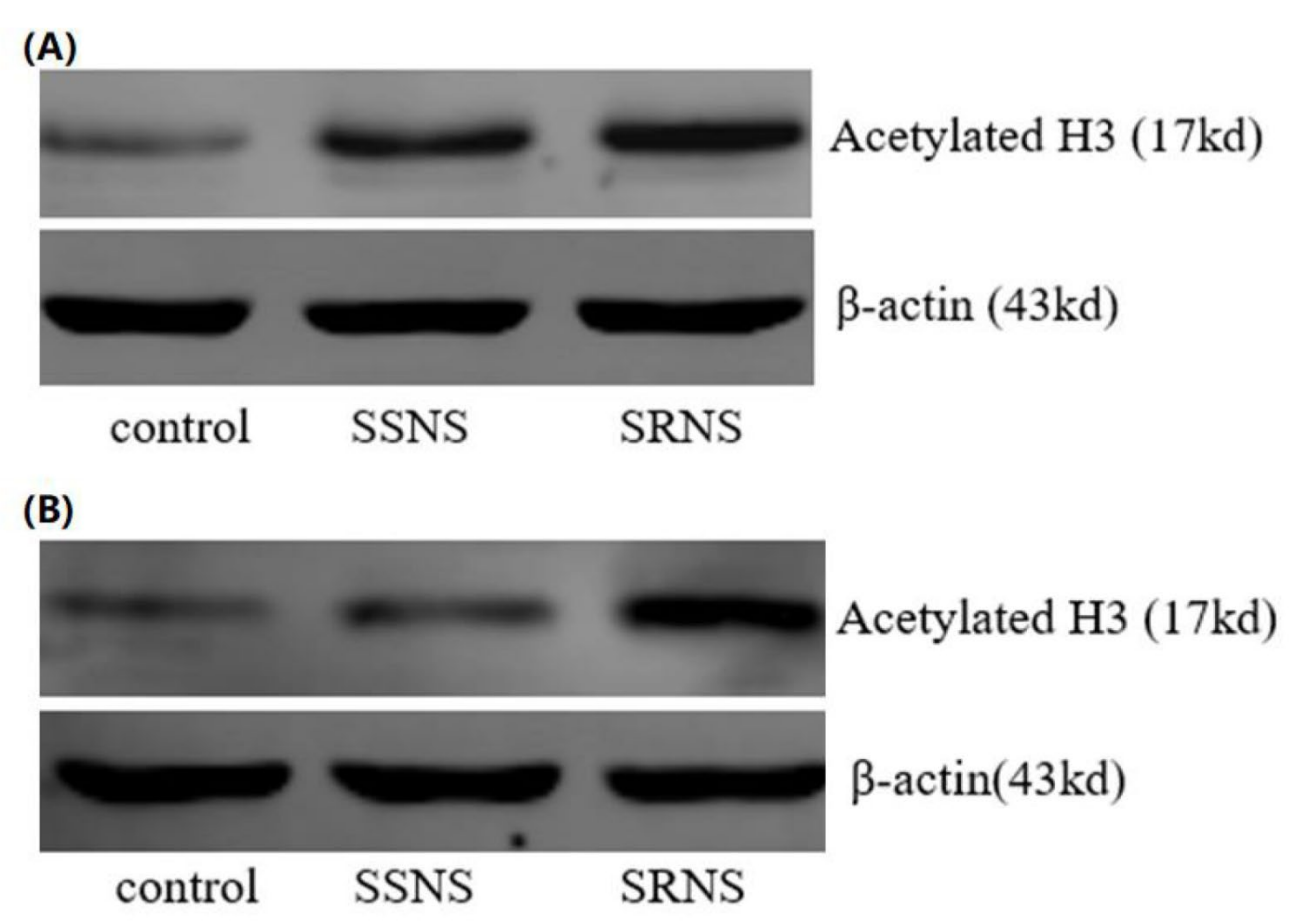
Acetylated H3 expression before (**A**) and after (**B**) the GC treatment. The acetylated H3 expression in the SSNS and SRNS groups is higher than that in the control before the GC treatment. The acetylated H3 expression in the SSNS and control groups is lower than that in the SRNS group after the GC treatment. a positive correlation between the NF-κB activity and the Ac-GR protein expression after the GC treatment was found in the SSNS group, in contrast to the negative correlation shown in the SRNS group. The opposite correlations between the NF-κB activity and the Ac-GR protein expression becomes pronounced for the difference in the two groups. A positive correlation for the difference between the Ac-H3 and Ac-H4 expression in the SSNS group, whereas fail to identify such correlation in SRNS

**Fig. 3:**
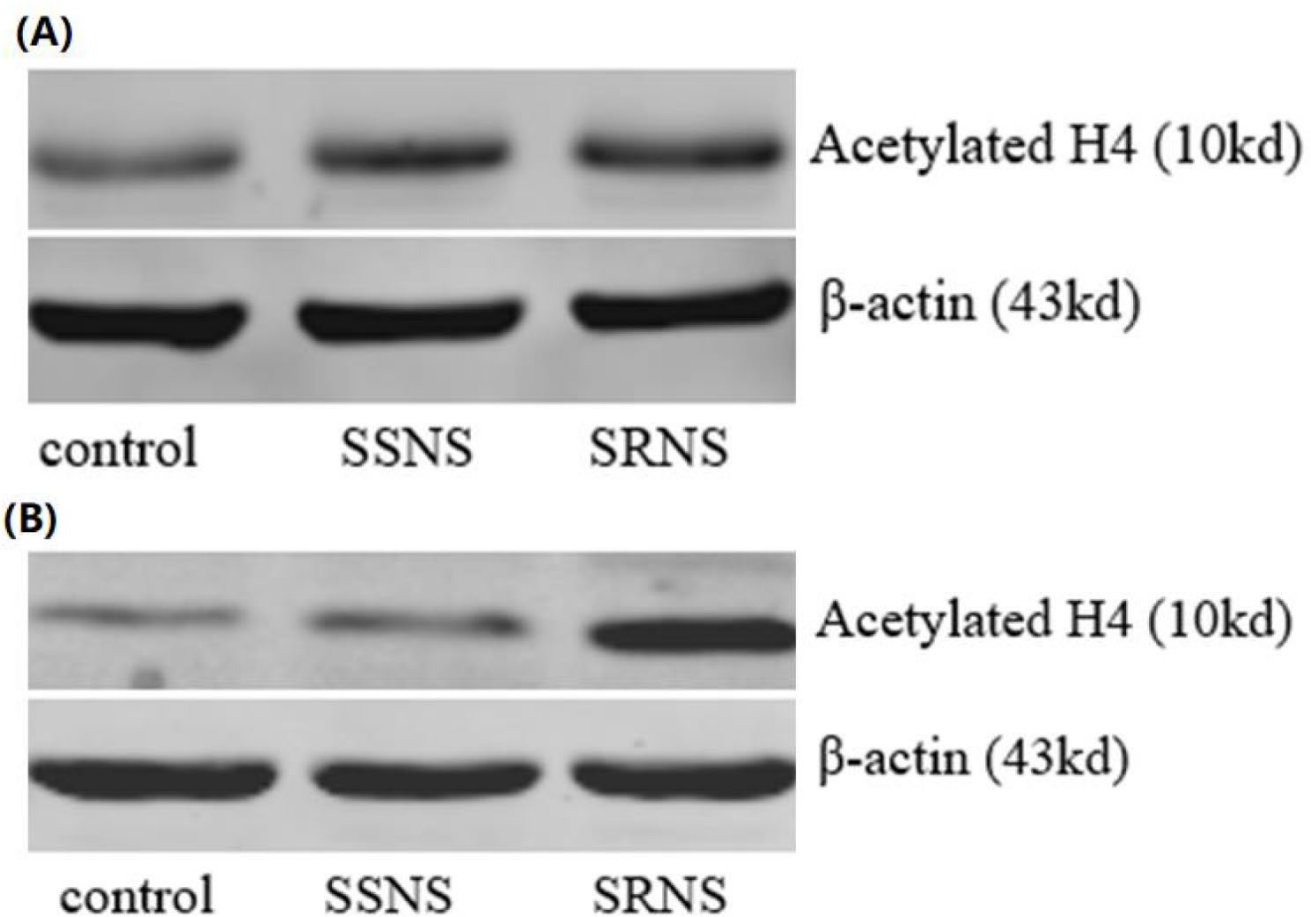
Acetylated H4 expression before (**A**) and after (**B**) the GC treatment. The acetylated H4 expression in the SRNS group was higher than those in the control and SSNS groups before the GC treatment. The acetylated H4 expression in the SSNS and control groups were lower than that in the SRNS group after the GC treatment

The expression levels of Ac-GR protein (Fig. 1A), Ac-H3 (Fig. 2A), Ac-H4 (Fig. 3A), and NF-κB activity exhibited significant differences among the three groups (Table 1). Notably, the Ac-GR protein was expressed at a relatively low level in the control group (mean: 0.331, *p* = 1.26E-4, *p* < 0.05 compared to SRNS and SSNS groups), at a significantly higher level in the SRNS group (mean: 0.498), and at a moderately high level in the SSNS group (mean: 0.429, with *p*-values indicating significant differences between SRNS and SSNS where applicable). Similar trends were also observed for the expressions of Ac-H3 and Ac-H4. Specifically, Ac-H3 levels were low in the control group (mean: 0.401, *p* = 3.04E-13, *p* < 0.05), elevated in the SRNS group (mean: 0.729), and moderately high in the SSNS group (mean: 0.652, with corresponding *p*-values). Similarly, Ac-H4 levels were low in the control group (mean: 0.324, *p* = 1.14E-16, *p* < 0.05), significantly higher in the SRNS group (mean: 0.664), and moderately elevated in the SSNS group (mean: 0.599, with relevant *p*-values). The NF-κB activity also followed this pattern, with low activity in the control group (mean activity level: 0.215, *p* = 8.51E-27, *p* < 0.05), high activity in the SRNS group (mean activity level: 0.576), and moderately high activity in the SSNS group (mean activity level: 0.397, along with the respective *p*-values indicating significance). All mean values and *p*-values are detailed in Table 1.

**Table 1:** Comparison of Ac-GR, Ac-H3 and Ac-H4 expressions and NF-κB activity before the GC treatment ($\:\stackrel{-}{\varvec{x}}$±s)

group (*n*)	Ac-GR	Ac-H3	Ac-H4	NF-κB
Control (15)	0.331 ± 0.066	0.401 ± 0.019	0.324 ± 0.034	0.215 ± 0.034
SRNS (15)	0.498 ± 0.113	0.729 ± 0.132	0.664 ± 0.100	0.576 ± 0.064
SSNS (32)	0.429 ± 0.107	0.652 ± 0.126	0.599 ± 0.098	0.397 ± 0.049
*F*	10.522	27.13	30.87	196.209
*p*	1.26E-4	3.04E-13	1.14E-16	8.51E-27

Note: The data are presented as mean ± standard deviation (± s). Analysis of variance (ANOVA) was performed to test for differences between the groups, with the *F*-value serving as the statistical measure for the ANOVA. The *p*-value is used to determine the statistical significance of the differences (a *p*-value < 0.05 is considered statistically significant)

### Comparison of Ac-GR, Ac-H3, Ac-H4 expressions and NF-κB activity after the GC treatment

The Ac-GR protein expression (Fig. 1B), Ac-H3 expression (Fig. 2B), Ac-H4 expression (Fig. 3B), and NF-κB activity after GC treatment were compared. Considering the baseline levels (i.e., the expressions prior to GC treatment), the differences for each expression were calculated, and the comparison analyses were conducted using the same statistical method as described above. It was found that, after GC treatment, the differences in expressions for all measurements were statistically significant across groups (Table 2). Specifically, for the SSNS children, the expressions of all four measurements decreased (*p* = 4.42E-11 for NF-κB, *p* = 8.41E-6 for Ac-GR, *p* = 5.38E-8 for Ac-H3, and *p* = 1.24E-7 for Ac-H4); whereas for the SRNS children, the expressions of all four measurements increased (*p* = 4.53E-5 for NF-κB, *p* = 8.81E-3 for Ac-GR, *p* = 0.510 for Ac-H3, and *p* = 0.132 for Ac-H4).

**Table 2:** Comparison of Ac-GR, Ac-H3, Ac-H4 expressions and NF-κB activity after the GC treatment ($\:\stackrel{-}{\varvec{x}}$±s)

group (*n*)	Ac-GR	Ac-H3	Ac-H4	NF-κB
Control (15)	-0.025 ± 0.104	0.017 ± 0.033	-0.012 ± 0.064	0.007 ± 0.070
SRNS (15)	0.173 ± 0.221	0.037 ± 0.213	0.076 ± 0.184	0.195 ± 0.130
SSNS (32)	-0.148 ± 0.157	-0.239 ± 0.190	-0.203 ± 0.168	-0.156 ± 0.090
*F*	19.52	18.32	19.08	68.79
*p*	3.12E-7	6.46E-7	4.07E-7	3.81E-16

Note: The data presented in this table compare the changes (Δ values) in the expression of acetylated glucocorticoid receptor (Ac-GR), acetylated histone H3 (Ac-H3), and acetylated histone H4 (Ac-H4), as well as NF-κB activity, after glucocorticoid (GC) treatment among the control group, steroid-resistant nephrotic syndrome (SRNS) group, and steroid-sensitive nephrotic syndrome (SSNS) group. The data are expressed as mean ± standard deviation (± s). The *F*-value represents the result of the analysis of variance, and the *p*-value indicates the significance level of differences between groups

### Correlation between Ac-GR, Ac-H3 and Ac-H4 expressions and NF-κB activity

**Fig. 4:**
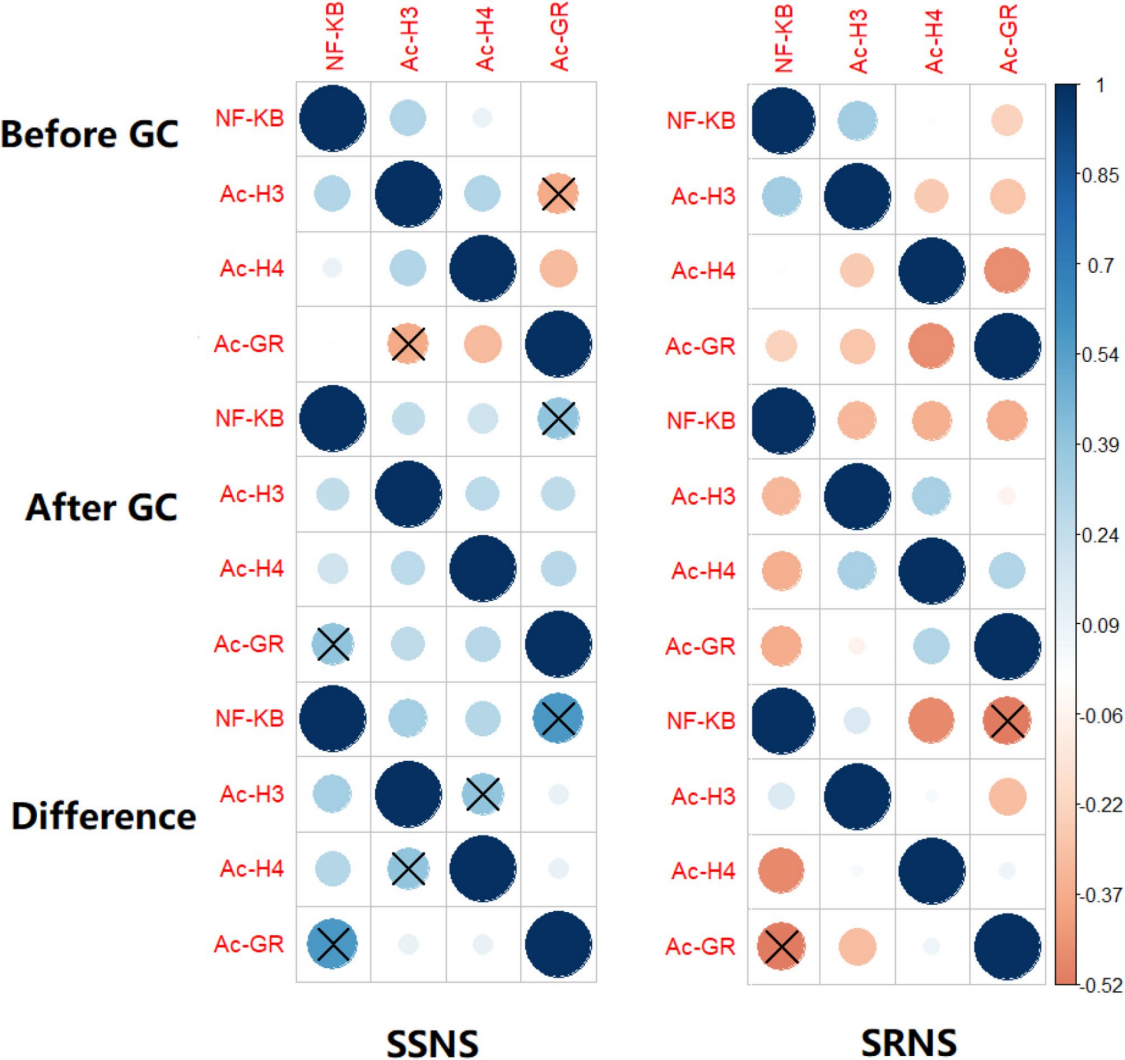
Correlation between of the NF-κB activity, the Ac-GR protein expression, Ac-H3 expression and Ac-H4 expression. The correlation is performed separately in the SSNS and SRNS groups and the significant correlations at the level of 0.05 are highlighted with a cross

The correlations among the four expressions before and after GC treatment were analyzed. Distinct correlations were observed between the SSNS and SRNS groups (Fig. 4). Notably, in the SSNS group, a positive correlation (*r* = 0.392, *p* = 0.026) was identified between the NF-κB activity and the Ac-GR protein expression after GC treatment, in contrast to the negative correlation (*r* = -0.367, *p* = 0.178) in the SRNS group. The disparity in the correlations between the NF-κB activity and the Ac-GR protein expression was more pronounced when considering the differences between the two groups (*r* = 0.574 with *p* = 5.96E-4 for SSNS vs. -0.519 with *p* = 0.047 for SRNS). Additionally, a positive correlation (*r* = 0.394, *p* = 0.026) was detected for the difference between the Ac-H3 and Ac-H4 expressions in the SSNS group, while no such correlation was found in the SRNS group.

### Mediation role of NF-κB activity in regulating Ac-GR, Ac-H3, and Ac-H4 expressions

In the current study, NF-κB activity was designated as the mediator variable, GC response as the exposure variable, and the expressions of Ac-GR, Ac-H3, and Ac-H4 as the outcome variables in the mediation analysis. It was observed that the GC response was significantly associated with the expressions of Ac-H3 (*p* = 5.25E-5) and Ac-H4 (*p* = 5.75E-6), but not with the expression of Ac-GR (*p* = 0.455). Furthermore, it was found that the GC response was highly significantly associated with the expression of NF-κB (*p* = 4.32E-14). The total effect, mediation effect, and direct effect are presented in Table 3. Notably, the direct effect of the GC response on the Ac-H4 expression remained significant after accounting for the influence of NF-κB expression (*p* = 0.007). Additionally, it was determined that the mediation effect of the GC response on the Ac-H3 expression was marginally significant (*p* = 0.083) and accounted for approximately 62% of the total effect relative to its indirect effect, suggesting that the GC response may influence the Ac-H3 expression indirectly through altering the expression of NF-κB. However, apart from the Ac-H3 expression, no significant mediation effect of the GC response on the Ac-GR expression or the Ac-H4 expression was identified.

**Table 3:** Mediation role of the NF-κB activity on the Ac-GR, Ac-H3 and Ac-H4 expressions

outcomes	a (se, *p*)	b (se, *p*)	c’ (se, *p*)	c (se, *p*)	ab (se, *p*)
Ac-GR	-0.352 (0.033, 4.32E-14)	0.169 (0.248, 0.680)	0.019 (0.0103, 0.856)	-0.041 (0.054, 0.455)	-0.059 (0.087, 0.498)
Ac-H3	-0.352 (0.033, 4.32E-14)	0.487 (0.277, 0.086)	-0.105 (0.115, 0.363)	-0.277 (0.062, 5.25E-5)	**-**0.171 (0.099, 0.083)
Ac-H4	-0.352 (0.033, 4.32E-14)	-0.052 (0.251, 0.838)	-0.297 (0.104, 0.007)	-0.279 (0.054, 5.75E-6)	0.018 (0.088, 0.837)

Note: *a* is the effect of the GC response on the NF-κB activity; *b* is the effect of the NF-κB activity on the Ac-GR protein expression, Ac-H3 expression or Ac-H4 expression; *c*’ is the direct effect of the GC response on the Ac-GR protein expression, Ac-H3 expression or Ac-H4 expression while adjusting for the NF-κB activity; *c* is the total effect of the GC response on the Ac-GR expression, Ac-H3 expression or Ac-H4 expression without adjusting for the NF-κB activity; *ab* is the meditation effect and is of our main interest, *se* and *p* in the parentheses are the standard error and the *p* value

## Discussion

As a transcription factor that is activated as a target during the action of GC, GR is highly relevant to SRNS. As a nuclear transcription factor, there exists an acetylation modification site within the DNA binding domain of the GR structure [27]. Our findings demonstrated that the level of acetylated GR in the SRNS group was significantly higher than that in the SSNS and control groups, indicating a potential role of the acetylated GR level in GC resistance. The level of acetylated GR in the SSNS group after treatment was lower than that prior to treatment, suggesting an association between acetylated GR and the adverse effect of GC in PNS children. Furthermore, we also observed that the acetylation level of GR in the SSNS group was lower than that before GC treatment, while in the SRNS group, it was higher than that before treatment. This implies that GC may act on GR acetylation, thereby resulting in different clinical responses to GC in PNS children during GC treatment and suggesting a relationship between GC response and the expression level of acetylated GR. Additionally, our study disclosed that the activity of NF-κB might mediate the impact of the GC response on the expression of Ac-H3.

In eukaryotic cells, genomic DNA associates with histone proteins, which are composed of four core histones (H2A, H2B, H3, and H4) and one linker histone (H1) [28]. Our results revealed that the levels of acetylated H3 and H4 in the SRNS group were remarkably higher than those in the SSNS and control groups. These findings suggest that the acetylation of H3 and H4 in the SRNS may enhance the expression of certain specific genes, which might possess a negative regulatory activity and contribute to GC resistance. Intriguingly, in the SSNS group (after eliminating the effect of GC), we noted significant correlations between NF-κB and H3/H4, indicating that disease conditions could potentially act on H3 or H4 by upregulating the expression of NF-κB in PNS children. Moreover, the current study also demonstrated that the levels of Ac-H3/H4 in the SSNS group after GC treatment were lower than those before treatment. This implies that, following the combination of GC and GR, GC might exert an influence on SSNS patients by modulating the acetylation of H3 and H4. The above results suggest an association between abnormal levels of Ac-H3 and Ac-H4 and GC resistance in PNS children, and also indicate that GC may impact acetylated histones. Some researchers have also reported that GC increases DNase I hypersensitivity, reduces nucleosome density, and augments the acetylation of histone H3 and H4 within the genomic regions surrounding the GR response element [29].

Another study has reported that a decreased GC response occurs in patients with severe asthma, asthmatics who smoke, all patients with COPD, and nephrotic syndrome. Several molecular mechanisms underlying GC resistance have been identified, which involve phosphorylation and other post-translational modifications of the GR protein. GC administration modifies the expression of HDAC molecules, thereby resulting in diverse responses to GC. HDAC2 exhibits a significant reduction in both activity and expression due to oxidative/nitrative stress and PI3 kinase-δ inhibition, ultimately leading to inflammation becoming resistant to the anti-inflammatory effect of GC. Initially, GC binds to GR in the cytoplasm and then translocates into the cell nucleus to inhibit the histone acetylation of NF-κB, which is a crucial nuclear factor involved in gene expression induced by certain cytokines. At the molecular level, GC interacts with cytoplasmic glucocorticoid receptor alpha (GCRα), triggering the translocation of GCRα to the nuclei of target cells. Activated GCRα interacts with coactivator complexes to induce histone H4 acetylation and subsequent transactivation, involving histone deacetylases (HDACs), especially HDAC2, to inhibit transcription [30].

The current research demonstrated that the activity of NF-κB was more pronounced in the SRNS group compared to the SSNS group. It has also been shown that the GC response exerts an effect on the expression of NF-κB, which is a conclusive finding in most related studies [31]. Our study also revealed that there was a direct effect of the GC response on the expression of Ac-H4 when adjusting for the expression of NF-κB, indicating that GC treatment might act on Ac-H4 expression. In our study, an evident total effect of the GC response on the expression of Ac-H3 or Ac-H4 was observed without adjusting for the expression of NF-κB, suggesting that in PNS patients, GC treatment could potentially affect the expression of Ac-H3 and Ac-H4 without the involvement of NF-κB. The elevation in the levels of Ac-H3, Ac-H4, and Ac-GR might be implicated in GC resistance in PNS children.

There is an increasing prevalence of GC resistance in PNS children, and the optimal therapeutic strategy for PNS children with GC resistance is to enhance their sensitivity to GC. Hence, our study has obtained suggestive evidence indicating that reversing GC resistance by reducing the levels of Ac-GR and histones might represent a promising clinical treatment approach.

There are some limitations of this research. In this study, the sample size was limited due to various conditions including limited samples; additionally, due to the incomplete data when the patients came for re-examination, the observation time points during the treatment were not enough; the detection of acetylation sites was also limited because of less blood volume.

## Conclusions

In this study, we emphasize that Ac-GR, Ac-H3, and Ac-H4 may exert certain effects on the GC response in children with PNS, and the acetylation of GR, H3, and H4 may be directly affected by GC administration. However, the specific mechanism requires further investigation, which is conducive to the discovery of noval therapeutic targets.

## Data Availability

The data that support the findings of this study are available from the corresponding author upon reasonable request.

## Abbreviations

GC: Glucocorticoid
Ac-GR: Acetylated GC receptor
Ac-H3: Acetylated histone3
Ac-H4: Acetylated histone4
NF-κB: Nuclear factor-κB
SRNS: Steroid-resistant nephrotic syndrome
SSNS: Steroid-sensitive nephrotic syndrome
PNS: Primary nephrotic syndrome
GR: Glucocorticoid receptor
HDACs: Histone deacetylases
COPD: Chronic obstructive pulmonary disease
ISKDC: International Study of Kidney Disease in Children
PBS: Phosphate buffered saline
HAT: histone acetylation transferase
GCRα: Glucocorticoid receptor alpha
